# Covid 19 and diabetes in children: advances and strategies

**DOI:** 10.1186/s13098-024-01267-2

**Published:** 2024-01-29

**Authors:** Zhaoyuan Wu, Jinling Wang, Rahim Ullah, Minghao Chen, Ke Huang, Guanping Dong, Junfen Fu

**Affiliations:** Department of Endocrinology, Children’s Hospital, Zhejiang University School of Medicine, National Clinical Research Center for Child Health, Hangzhou, China

**Keywords:** COVID-19, Diabetes Mellitus, Type 1, Child

## Abstract

Throughout the COVID-19 pandemic, there has been a notable increase in the incidence of new-onset diabetes and diabetic ketoacidosis (DKA). Simultaneously, children diagnosed with type 1 diabetes (T1D) have encountered difficulties in maintaining optimal blood glucose levels. The mechanisms underpinning these correlations still remain a puzzle. We reviewed the studies that examined changes in incidence during the pandemic. These studies utilized various metrics for comparison, which encompassed the timing of data collection, diagnostic criteria, as well as the numbers and incidence rates of diabetes and DKA. We found the incidence of diabetes and DKA was higher during the pandemic. As to mechanisms, the invivo and invitro study revealed the factors such as direct viral damage, metabolic dysfunction, and immune responses all attribute to the process of T1D after suffering from COVID-19. Furthermore, we provide some useful strategies to prevent and treat children suffering from diabetes and COVID-19. Conclusions: Strong correlations have been observed between new-onset diabetes and COVID-19. Insights gleaned from clinical descriptions and basic research can offer valuable experience and recommendations for the treatment and prevention of diabetes during future pandemics.

## Introduction

Since 2019, COVID-19 has spread globally, with nearly 771,820,937 confirmed cases and 6,978,175 deaths worldwide [1]. Among its complications, diabetes has garnered increasing attention due to its association with heightened mortality risk. Moreover, there have been numerous reported cases worldwide of children developing new-onset diabetes after contracting COVID-19 [2, 3]. Hence, it is imperative to establish the causal link between COVID-19 and new-onset diabetes in children. While extensive efforts have been made to unravel the potential mechanisms of this infectious disease, encompassing viral transmission, replication, and damage to endocrine tissues [4], the relationship between COVID-19 and diabetes remains enigmatic. In clinical practice, children with diabetes face challenges in managing their blood glucose levels during the pandemic [5], while those in intensive care units necessitate sound judgment and adherence to standardized treatment protocols when dealing with virus infections complicated by DKA.

Given the impact of COVID-19 infection on the incidence of new-onset diabetes, there also remain puzzles that need to be solved. In epidemiology, the characteristics of children with new-onset diabetes after infection are predominantly derived from single centers or case reports. However, there is a pressing need for evidence showing the rising incidence of diabetes and DKA across multiple centers. Besides, as the incidence of diabetes and COVID-19 among children continues to rise, the causal relationships of new-onset diabetes after infection lack clear clues. It is vital and urgent to comprehensively summarize and identify associations, as this could unveil potential targets for further treatment and prevention. Moreover, the characteristics of new-onset diabetes after infection have not been fully elucidated, potentially leading to diagnostic errors. In the face of limited clinical resources, healthcare professionals and parents are confronted with challenges in treating DKA following COVID-19 infection and managing glucose levels in children with diabetes, and the experiences and suggestions gathered from various guidelines and reports become crucial and urgently needed for clinical practice.

In this review, we comprehensively analyze epidemiological studies conducted since the onset of the COVID-19 pandemic to elucidate the evolving trends in pediatric diabetes during this crisis. Drawing from both in vivo and in vitro research, we consolidate the potential mechanisms through which viral infections may lead to diabetes. In the third section, we compile practical suggestions for blood glucose monitoring and treatment of children experiencing DKA in the context of viral infections. Additionally, we endeavor to provide prospects for the research between COVID-19 and new-onset diabetes.

## Epidemiology

### Post-COVID-19 infection may elevate the risk of hyperglycemic states

Numerous studies have examined the incidence of type 1 diabetes (T1D) in children before and after the COVID-19 pandemic, as detailed in Table 1. Compared to the incidence of diabetes before the pandemic, there was a significant increase after the COVID-19 outbreak. Notably, DKA, a severe condition associated with diabetes, has become more frequent during the pandemic One of the most extensive international multicenter studies conducted to date was led by Niels H. Birkebaek. Drawing upon data from 13 national diabetes registries, the research revealed a significantly elevated observed prevalence of DKA at the time of T1D diagnosis compared to the predicted prevalence based on the previous decade’s data [6]. Another multicenter regional study conducted in the UK revealed a higher proportion of children presenting with DKA in the COVID-19 PCR-positive groups [2]. A study conducted in Canada, based on data from the pandemic period of March 17, 2020, to August 31, 2020, reported a significantly higher frequency of DKA at the onset of T1D during the pandemic, with an increased incidence of severe DKA [7]. In a study involving 13 international centers, scientists also asserted that the adjusted observed prevalence of diabetic ketoacidosis in 2021 was significantly higher than the predicted prevalence for that year [6]. Similar findings emerged from studies conducted in many countries [3, 7–16]. Conversely, some researchers reported contrasting findings [17, 18], their conclusion, however were based on small-populations or restrict-range study which may reduce their reliability.

**Table 1:** Studies on Changes in the Incidence of New-Onset Diabetes and Diabetic ketoacidosis (DKA) in Children during the Pandemic. (PICU, Pediatric Intensive Care Unit; T1D, type 1 diabetes; NR, not reported)

General characteristics of epidemiological studies during the pandemic vs. before										
Study	Design	Country	Age	Gender, (M)	Data collection period during the pandemic	T1D diagnosis criteria	DKA diagnosis		Outcome	
								Group	before	after
Ivana Rabbone(17)	Cross-sectional	Italian	0– 14	NR	20 February 2020-14 April 2020	Autoantibody positivity	International Society for Pediatric and Adolescent Diabetes criteria	Diabetes	208	160
								DKA	86	61
								Severe DKA	31	27
Clemens Kamrath(3)	Cross-sectional	Germany	9.9 (5.8–12.9)	327 (61.5)	13 March 2020-13 May 2020	NR	DKA: pH level less than 7.3 and/or bicarbonate level less than 15 mmol/L severe: a pH level less than 7.1 and/or bicar- bonate level less than 5 mmol/L	DKA	24.50%	44.70%
								Severe DKA	13.90%	19.40%
Heli Salmi(61)	Retrospective cohort study	Finland	10.0 (8.1–12.3)	11 (55)	1 April 2020–31 October 2020	NR	Blood pH < 7.10	PICU-DKA	6.25	20
								PICU-Severe DKA	19	15
								Cohort-DKA	57.75	84
								Cohort-Severe DKA	20	13
Josephine Ho(7)	Retrospective chart review	Alberta, Canada	less than 18 years	46 (43)	17 March 17 2020-31 August 2020	2018 Diabetes Canada Clinical Practice Guidelines	Hyperglycemia (blood glucose > 11 mmol/L) and blood pH < 7.3 or bicarbonate < 15 mmol/L and ketonemia or ketonuria (based on beta-hydroxybutyrate level or pres- ence of urinary/serum ketones)	Diabetes	114	107
								DKA	52	73
Sinéad M. McGlacken-Byrne(89)	Cross-sectional study	North Central London	10.6 (range 3.2–16.3)	9 (52.9)	23 March 2020 − 30 June 2020	NR	A pH level less than 7.3 and severe DKA as a pH level less than 7.1	No DKA	21	4
								Mild DKA	4	1
								Moderate DKA	2	4
								Severe DKA	3	8
Niels H Birkebaek(6)	International multicentre study	International	9·9 (6·1–12·8)	4521 (55.1%)	2020	NR	International Society for Pediatric and Adolescent Diabetes (ISPAD) criteria	DKA	27.30%	36.60%
Shira Goldman(90)	Population-based study	Israeli	9.9 7.5,13.2)0.2]	87 (59.6)	15 March 2020-30 June 2020	ISPAD	pH level less than 7.3 or a bicarbonate level less than 15 nmol/L	Diabetes	113	146
								Mild DKA	17	27
								Moderate DKA	12	29
								Severe DKA	15	29
Arzu Jalilova(91)	Cross-sectional study	Turkey	9.4 (0.8–18)	105 boys (52.7%)	March 2020-March 2021	ISPAD	ISPAD criteria	DKA	55.30%	58.30%
								Severe DKA	24.20%	30.40%
Mikael Knip(16)	Observational cohort study	Finland	8·41 (3.75)	458 (58·3%; 54·9 to 64·7%)	1 March 1 2020-31 Aug 2021	American Diabetes Association (ADA) criteria	NR	DKA	22.60%	30.80%
								Severe DKA	5.90%	8.80%
C. Baechle(8)	Cross-sectional study	Germany	9.8 (6.0–12.9)	55.90%	2020	NR	DKA was defined as pH-value < 7.3 and/or bicarbonate level < 15 mmol/l Severe DKA (sDKA) as pH-value < 7.1 and/or serum bicarbonate level < 5 mmol/l	Diabetes	19.80%	24.90%
								DKA	4.10%	8.30%
								Severe DKA	1.40%	3.00%

Masoud Rahmati and colleagues conducted a random-effects meta-analysis on the relationship between COVID-19 and diabetes. Their analysis revealed that the COVID-19 pandemic led to a 9.5% increase in global new-onset T1D cases among pediatric populations, the global number of children experiencing DKA and severe DKA increased by 9.5%, 25%, and 19.5%, respectively, when compared to pre-COVID-19 years [19]. In separate meta-analysis from other countries and time periods, it was also demonstrated that the risk of new-onset T1DM and DKA following COVID-19 infection in children and adolescents was higher compared to non-COVID-19 control groups [20–22].

### Children with diabetes facing heightened risks during COVID-19

#### High glycemia level may lead to bad outcome after COVID-19

Increased COVID-19-related mortality has been linked not only to cardiovascular and renal complications of diabetes but also to glycemic control and body mass index. Several studies have indicated that adults with T1D face an elevated risk of COVID-19 complications. For instance, scientists discovered a correlation between elevated Glycosylated Hemoglobin, Type A1C (HbA1c) levels and increased COVID-19-related mortality and hospitality [23, 24]. Additionally, research involving adult diabetes patients has identified hyperglycemia as an independent factor associated with a severe prognosis in individuals hospitalized for COVID-19 [25, 26]. Conversely, some studies have reported contradictory findings. Roque Cardona-Hernandez and colleagues revealed that children with diabetes exhibited similar outcomes and prognosis to their non-diabetic peers across five centers [27]. Similarly, Revital Nimri and his colleagues found that young individuals with established T1D experienced mild COVID-19 infections in Israel. However, their research also suggested that elevated glucose levels during COVID-19 infection and older age were associated with a prolonged disease course [28].

#### Individuals with T1D encountered heightened challenges in managing their blood glucose levels

The number of T1D children has increased sharply in recent years [29], how to face the risks from pandemic is a challenge for patients and their parents. A cross-sectional study involving 98 children and adolescents with T1D identified several key challenges faced by Iranian patients due to pandemic restrictions. These challenges included the necessity for increased insulin doses, reduced physical activity, insulin shortages, and missed morning insulin doses [30]. Other studies also noted weight gain, a significant rise in HbA1c levels, and an increase in daily insulin requirements among children with T1D during the pandemic [31, 32]. Furthermore, the mental health of children with diabetes has gained increased attention. A retrospective evaluation of 39 patients with T1D who experienced acute hospitalizations revealed that approximately 6 out of 11 (55%) patients hospitalized in 2021 had witnessed diabetes deterioration due to emotional distress, a phenomenon not prominently observed in the pre-COVID era [33]. Additionally, S. B. Koca and colleagues conducted a cross-sectional clinical and laboratory study, revealing a statistically significant positive correlation between HbA1c levels and Children’s Depression Inventory (CDI) scores, which serve as an indicator of depression status [34].

Conversely, María Sánchez Conejero and her colleagues reported contrasting results in an observational, retrospective study involving children and adolescents with T1D who utilized interstitial glucose monitoring systems. Their findings indicated that during a 2-week lockdown period, the glycemic control of children with T1D actually improved, particularly among those who had poorer baseline control [35]. Interestingly, another observational cohort study conducted by Namam Ali suggested improvements in glucometer data, including HbA1c and data from finger glucose monitoring (FGM) systems, among individuals with T1D during the COVID-19 pandemic and lockdown. Notably, FGM users demonstrated better outcomes [36]. Furthermore, dietary management has become increasingly crucial in diabetes treatment. A study involving 764 participants highlighted that eating habits were significantly worse among youths with diabetes compared to those without diabetes. This underscores the importance of lifestyle education during the pandemic [37].

## Mechanistic interaction between COVID-19 and diabetes

### Direct damage to pancreas

#### Viral infection and diabetes

Numerous reports and studies have explored the connection between viral infections and the onset of new diabetes cases. A compelling body of evidence has emerged from cases of robust type-1 diabetes characterized by the sudden onset of insulin-dependent hyperglycemia and, in some instances, ketoacidosis, even in individuals without detectable autoantibodies. Symptoms such as fever and cough have been identified as potential triggers for this new-onset diabetes. Virology studies have further detected RNA from enteroviruses in four patients afflicted with new-onset diabetes. Meta-analysis have provided substantial evidence linking enterovirus infections to the significant incidence of new-onset diabetes [38]. Furthermore, researchers have observed that individuals infected with Hepatitis C face an elevated risk of developing diabetes when compared to non-infected populations [39]. In vitro studies have also demonstrated that Coxsackie B virus can lead to beta cell dysfunction or even cell death [40].

During the acute phase of viral infection, the virus can replicate within pancreatic β cells, resulting in the direct destruction of these cells and triggering a cytotoxic immune response [41]. Following the acute phase, β cells within the islets are predominantly targeted by autoimmune processes and epitope diffusion. Sustained overexpression of major histocompatibility complex (MHC) molecules can expose β cell epitopes to the immune system, heightening the risk of autoimmune dysfunction. Another crucial mechanism of damage involves bystander effects, which are mediated by cytokines, particularly interferon-alpha (INF-α), interferon-beta (INF-β), interferon-gamma (IFN-γ), tumor necrosis factor (TNF), and interleukin-1beta (IL-1β). These cytokines can induce endoplasmic reticulum stress and trigger apoptosis in islet cells, as observed in animal models. Additional potential mechanisms include molecular mimicry and bystander activation, although their relevance to humans remains to be conclusively established.

#### The mechanisms of covid-19 cellular entry

**Fig 1:**
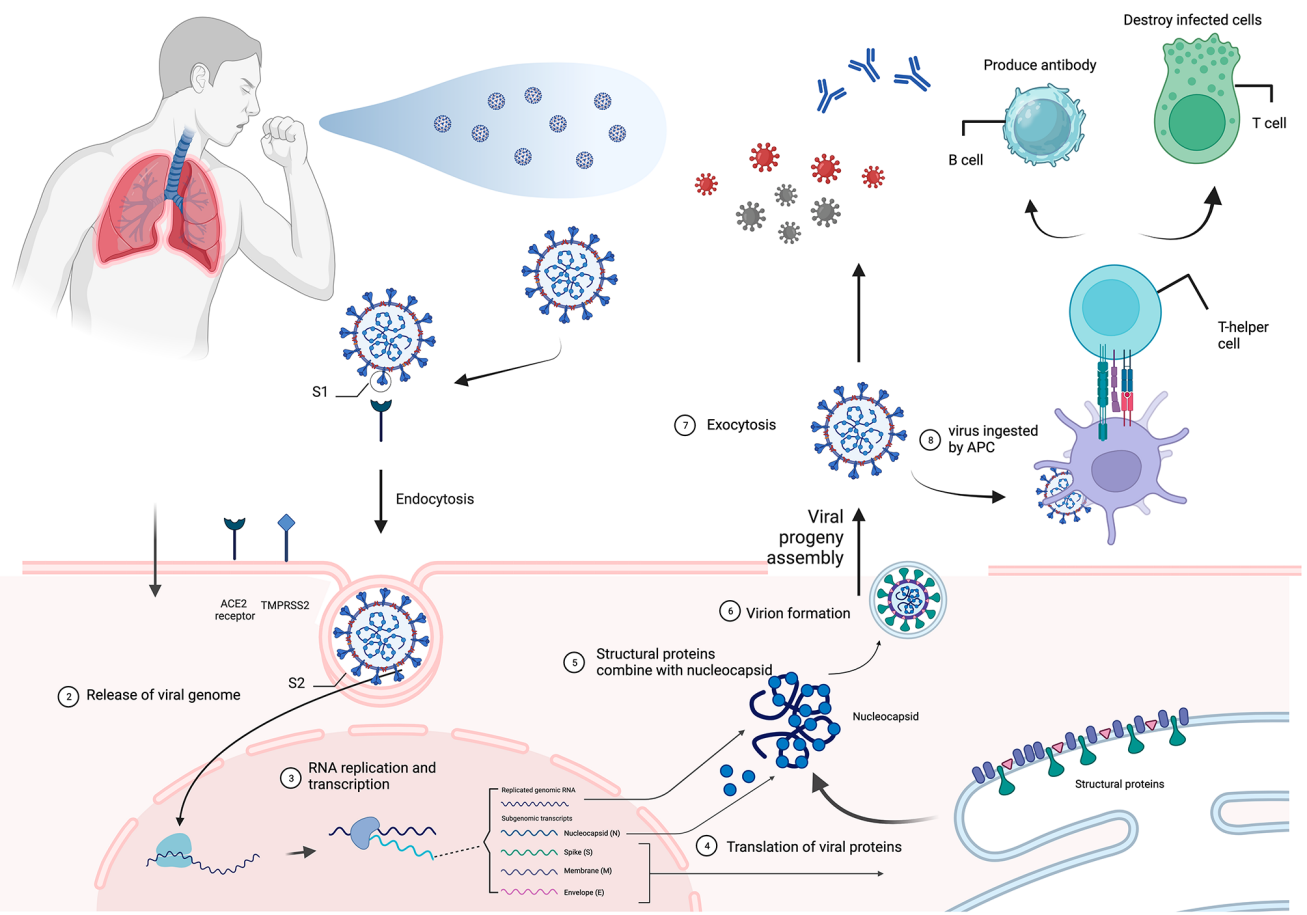
The fundamental infection process triggered by COVID-19 and subsequent immune response in the human body. (APC, Antigen-presenting cells; S1, spike 1;S2, spike 2; ACE2, angiotensin-converting enzyme 2; B cell, bursa dependent lymphocyte; T cell, thymus dependent lymphocyte)

The infection process is illustrated in Fig. 1. COVID-19, a positive-sense single-stranded RNA coronavirus, comprises four essential protein components: membrane (M), envelope (E), nucleocapsid (N), and spike (S) proteins. During the cellular entry process, the S protein interacts with angiotensin-converting enzyme 2 (ACE2), which is widely expressed in the human respiratory system. Subsequently, the virus undergoes endocytosis, leading to the fusion of the virus with the host cell membrane. Simultaneously, the bound S protein is cleaved by the membrane-bound serine protease Transmembrane Serine Protease 2 (TMPRSS2), activating the endocytic process and enabling the virus to enter the host cell. In this context, the RNA within the virus directly engages with ribosomes to initiate replication and translation processes, resulting in the production of viral proteins and RNAs. Following modification, assembly, and packaging, the virus is released from the host cell through exocytosis, subsequently infecting other cells.

### COVID-19 infection of pancreatic beta cells

#### Evidence from clinical samples from human

Pancreatic β cells, responsible for insulin synthesis and secretion, play a pivotal role in regulating glucose levels within the human body. In the context of COVID-19 infection, the ACE2 receptor assumes a central role. Analyses of public databases have demonstrated the expression of ACE2 receptors not only in exocrine glands but also in islets, including β cells, with higher expression levels compared to the lungs [42]. Besides, the expression of ACE2, TMPRSS, Dipeptidyl peptidase 4 (DPP4), high molecular group box 1 (HMBG1), and Neuropilin-1 receptor (NRP1) on the membrane of islet β cells, also facilitates viral entry. The investigation into the impact of COVID-19 on islet β-cells initially stemmed from autopsy reports, where scientists observed pancreatic tissue degradation in three patients who succumbed to COVID-19 infection. A study in Wuhan, China, further identified elevated levels of amylase and lipase in COVID-19 patients, even in cases with mild symptoms, with more significant elevations seen in severe cases. Computed tomography scans of severely infected COVID-19 individuals indicated pancreatic changes such as pancreas enlargement or pancreatic duct dilation, without signs of acute necrosis [42]. Syairah Hanan Shaharuddin and colleagues conducted a study utilizing live human pancreatic cultures and postmortem pancreatic tissue from COVID-19 patients, revealing the virus’s ability to infect pancreatic tissues, encompassing endocrine islets, exocrine acinar cells, and ductal cells within the pancreas [43].

#### Structure and transcriptional change in islets after infection

In infected human islets, both insulin content and glucose-stimulated insulin secretion experienced significant decreases when compared to mock-treated islets, implying a detrimental impact on islet function. Janis A Müller and colleagues found following infection, endocrine cells exhibited subcellular and functional damage, including dilation and vacuolization of the endoplasmic reticulum (ER)-Golgi apparatus complex, as well as a loss of the ability to transition from high glucose levels to low glucose levels [44]. Transcriptional analysis revealed defects in protein secretion, a loss of cell identity, and an enrichment of IFN type 1 response [44]. Additionally, in another study, by staining for autopsy tissue from COVID-19 patients and normal individuals, the scientists suggested that COVID-19 infiltration induces the expression of phosphorylation of mixed linage kinase domain-like protein (pMLKL), which may be considered a signal of necroptosis [45]. Besides, scientists observed that infection induced apoptosis in β-cells. Through the application of global phosphoproteomics, substrate-based kinase activity prediction, along with kinase set enrichment analysis and gene set enrichment analysis, they unveiled an upregulation of stress-response Mitogen-activated protein (MAP) kinases, including c-Jun NH2-terminal kinase (JNK)/p38 (MAPK8/11), and cytoskeleton-reorganizing p21-activated kinases (PAK), both of which can trigger cell death through apoptosis pathways. Furthermore, several members of the protein kinase C (PKC) family exhibited downregulation [4].

### Damage from inflammation induced by infection

Moreover, as a pathogen, COVID-19 can trigger a cytokine release syndrome, characterized by elevated levels of inflammatory cytokines, including IL-1β [46], interleukin 1 (IL-6) [47], interleukin 12 (IL-12) [48], IFN-γ [49], and TNF-α [50]. These inflammatory cytokines may result in β cell dysfunction and damage, as well as stimulate immune cell responses within the islets, ultimately leading to cell destruction via intrinsic cellular signaling pathways. Some scientists have also suggested that COVID-19 infection can induce a hypercoagulable state, causing endothelial injury, which in turn exacerbates microvascular inflammation, thrombosis, and damage to cells in human islets [51].

#### Susceptibility in islets cells under infection

In pancreatic tissues, there exist two types of islet cells, β cell plays a more important role in glucose metabolism, however, is more vulnerable in damage of infection. In a separate study that centered on pancreatic tissue from donors who had not been infected by COVID-19, scientists discovered that ACE2 and TMPRSS2 were generally expressed in both β-cells and α-cells but at low protein levels. However, two other receptors, NRP1 and transferrin receptor protein 1 (TFRC), displayed robust expression specifically in β-cells, not in α-cells. This suggests a potential mechanism for COVID-19’s tropism for β-cells [4]. In another in-vitro study, the efficiency of COVID-19 infection significantly decreased in the following co-treatment with the small molecule EG00229, a selective NRP1 antagonist [4].

### Dysfunction beyond pancreatic

**Fig 2:**
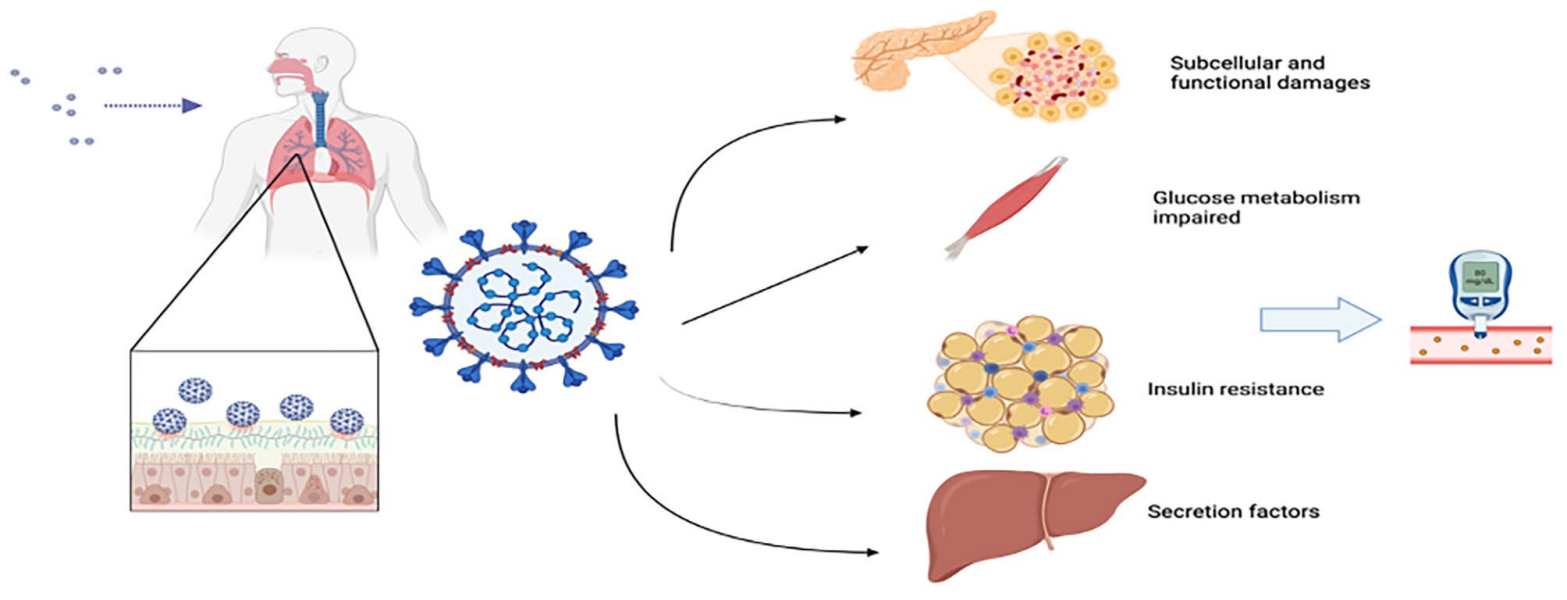
The dysfunction of endocrine organs and its impact on metabolic systems following COVID-19 infection

In addition to directly affecting human islets, COVID-19 can also induce hyperglycemia by infecting other tissues, as illustrated in Fig. 2.

#### Glucose metabolism

Glucose metabolism plays a crucial role in the mechanisms of diabetes. Catabolic processes like glycolysis occur in the liver, muscle, and nearly all peripheral tissues, while anabolic stages include gluconeogenesis and glycogen synthesis. These pathways collectively regulate glucose levels within the body. Any impairment in glucose metabolism within these pathways can result in glucose level dysfunction. Multiple studies have identified elevated blood glucose levels as a significant independent risk factor for severe infection and worse outcomes. Ana Campos Codo and her colleagues conducted a study focusing on monocytes and macrophages, which are believed to play an essential role in the disease’s pathogenesis. They discovered that these cells undergo a shift towards heightened glycolysis after infection, facilitating COVID-19 replication [52]. In vitro studies further demonstrated that increasing glucose levels in culture led to a significant rise in viral load, as well as the expression of ACE2 and IL-1β in monocytes infected by COVID-19. Elevated glucose levels also trigger the secretion of cytokines, including TNF-α, IL-6, IFN α, β, and λ. Monocytes isolated from tissues of diabetes patients exhibited a higher viral load compared to normal controls [52]. Proteomic studies suggested an upregulation of glycolysis-related pathways after infection, indicating that increased glycolysis could be a distinctive feature of COVID-19 infection compared to other respiratory viruses [52]. Additionally, further research based on bronchoalveolar lavage (BAL) monocytes from severe COVID-19 patients found that metabolic state changes were promoted by hypoxia-inducible factor-1α (HIF-1α), which was stabilized by mitochondrial reactive oxygen species (ROS) production triggered by infection [52].

#### Inflammation

The most prevalent pathophysiological change observed in the organs of COVID-19 patients is uncontrolled inflammation. Key manifestations include widespread damage to the alveoli, infiltration of inflammatory cells in hyaline membranes, as well as inflammation in the liver, myocardium, and brain. Jérôme Hadjadj and colleagues conducted an integrated immune analysis on a cohort of 50 COVID-19 patients with varying disease severity. They discovered a significant impairment in the interferon (IFN) type I response, which was associated with persistent viral load in the bloodstream and an exacerbated inflammatory response. However, the inflammation is primarily driven by increased production and signaling of TNF-α and IL-6 [53]. In addition to changes in immune types, severe COVID-19 patients also experience cytokine storms, which can pose a life-threatening risk. A retrospective study conducted in Wuhan indicated alterations in immune types in patients following COVID-19 infection [47]. Furthermore, the researchers identified IL-6 and lactate dehydrogenase (LDH) as independent parameters for predicting the severity of COVID-19. In innate immunity, IL-6 plays a crucial role as a pro-inflammatory factor, capable of causing damage to proteins, lipids, and DNA in organs and tissues, ultimately affecting the body’s structure and function [54]. Elevated inflammatory biomarkers like IL-6 have been linked to an increased risk of both microvascular and macrovascular complications due to low-grade vascular inflammation, which could contribute to the severity of diabetes [55].

#### Insulin resistance

Insulin resistance plays a critical role in the development and progression of diabetes, particularly in type 2 diabetes. However, insulin resistance following COVID-19 infection differs from diet-induced insulin resistance, as it is primarily driven by inflammation affecting metabolic organs or the entire body. Elettra Barberis and her colleagues utilized metabolomic approaches to identify lipid biomarkers in COVID-19 patients, providing evidence of insulin resistance development post-infection [56]. Moritz Reiterer and his colleagues observed a frequent association between hyperglycemia and severe respiratory syndrome in COVID-19 patients, with insulin resistance identified as a leading factor. Their research also revealed lower levels of adiponectin in COVID-19 patients. Subsequent studies indicated that COVID-19 has the capacity to replicate in hamster adipose tissue and modify adipokine expression, particularly leading to a reduction in adiponectin expression [57].

In addition to adipose tissue, the metabolic condition of skeletal muscle also plays a role in the severity of insulin resistance. Marko Šestan and colleagues discovered that the virus-induced IFN-γ can reduce the expression of insulin receptors in skeletal muscle, resulting in a state of hyperinsulinemia. This state can further boost antiviral immunity by directly stimulating the function of CD8 + effector T cells. Consequently, viral infection may accelerate the development of diabetes in individuals with pre-existing insulin resistance conditions, such as obesity or fatty liver diseases [58].

#### Circulation

As a state of endocrine dysfunction, the interplay between organs holds significant implications for the development and severity of diseases. Endocrine hormones serve as crucial bridges between these organs. In light of the evidence indicating hyperglycemia following COVID-19 infection, researchers have identified a glucogenic hormone known as GP73 [59]. GP73 is secreted by various tissues during fasting and is notably elevated in lung, liver, and kidney tissues post COVID-19 infection. Experiments with cultured cells have shown that GP73 secretion increases following COVID-19 infection or overexpression of COVID-19 nucleocapsid and spike proteins in a dose and time-dependent manner [59]. Studies on mice have revealed that after GP73 injection, these molecules are primarily transported to the liver and kidneys, with higher accumulation observed after 30 min. Additionally, researchers have demonstrated that GP73 can enhance the expression of key gluconeogenic enzymes, intracellular cAMP levels, and PKA kinase activity, thereby increasing glucogenesis. Subsequent investigations have indicated that blocking GP73 can inhibit excessive glucogenesis and reduce elevated glucose levels during fasting stages after COVID-19 infection in vitro and in vivo [59]. As detection technologies advance, more hormones remain to be discovered, potentially serving as therapeutic targets in the future.

## The management advice in patients with COVID-19 and diabetes

During the COVID-19 pandemic, individuals with new-onset or pre-existing diabetes encountered significant challenges in managing their blood glucose levels. As we reflect on the lessons and experiences gained from the pandemic’s impact on diabetes, this section will outline advanced strategies for blood glucose control and offer clinical recommendations for managing new-onset diabetes in COVID-19-positive patients.

### The causal relationships between COVID-19 and diabetic ketoacidosis

During the pandemic, the incidence of DKA increased significantly compared to before. As a severe condition of T1D, the physiological causal relationships alone cannot fully explain this phenomenon. Other contributing factors need attention from doctors and parents. Firstly, with a sharp increase in children infected by the virus, there was a simultaneous exaggeration of the shortage of clinical resources. Children with diabetes might not be diagnosed promptly, leading to delayed insulin treatment and prolonged symptoms compared to previous years [60]. Additionally, due to lockdown policies, children with T1D may face difficulties accessing convenient clinical guidance, potentially resulting in poorer control of their blood glucose levels [61]. The application of telephone medical services in some countries and areas has proven effective and efficient in helping manage blood glucose levels [62]. Moreover, as COVID-19 is a viral infection, its symptoms may differ significantly. The signs of diabetes could be dismissed initially. Furthermore, COVID-19-induced cytokine storms, the use of steroids for COVID-19 treatment, and changes in lifestyle during the pandemic can all contribute to an increased incidence of new-onset DKA in children [60].

### Suggestions for children suffered from diabetes and COVID-19

As children suffer from COVID-19, doctors and families should be aware of the symptoms of diabetes. If the children feel more thirsty and hungry than before, urinate a lot than usual and lose weight without trying their blood glucose level, a comprehensive laboratory test is important and necessary [63]. As mentioned in literature, new-onset diabetes is processed aggressively during the pandemic and lockdown, doctors and families need to pay attention to the clinical signs of DKA, and provide positive and effective treatment for them [7]. Besides, doctors should monitor the plasma glucose, electrolytes, pH, and blood ketones of hydroxybutyrate to recognize severe cases immediately [64]. Furthmore, some effective monitor warning system for DKA also could be applied before comprehensive laboratory tests [65]. For prevention, multiple studies found the severity of infection will influence the risk of new-onset diabetes [66], and the application of vaccines could ameliorate the severity of infection [67]. Therefore providing positive treatment for infection and injecting vaccine will lower the rates of new-onset diabetes, although the conclusion needs more direct evidence [68].

Diabetes condition may lead to bad outcomes after COVID-19 infections [23]. For children with diabetes, preventing the infection of COVID-19 has great meaning. Italian Society for Pediatric Endocrinology and Diabetology (ISPED) recommends the children follow the following suggestions to lower the risks of infection: (1) Stay at home, and avoid crowded places. (2) Wash the hands, don’t touch eyes, nose and mouth (3) Cover mouth and nose with bent elbow or tissue when coughing or sneezing (4) Avoid contacts with other persons, particularly if affected by COVID-19 (5) Don’t interrupt vaccination program [69]. As to the children with T1D, they suggest the parents and children maintain good metabolic control, and increase the glucose monitoring by means of technological devices including pumps and continuous glucose monitoring. Besides, it is necessary to promote adherence to healthy nutrition, increase fruit and vegetable intake to avoid minerals and oligo-elements deficiency and reduce high-calorie food intake. Furthermore, they encourage the children to continue regular physical activity [69]. In case of suspected symptoms, including difficult breathing or shortness of breath, persistent pain and chest pressure, parents should immediately contact the general practitioner and the hospital.

### Managing DKA in the intensive care unit

Numerous studies have indicated that the incidence of DKA is associated with an increased risk of mortality from infections. Hence, early recognition of the clinical signs of DKA is crucial. Initial case reports described the clinical characteristics of children with both COVID-19 and diabetes. Common symptoms included weight loss, polyphagia, and polyuria [70], resembling the symptoms of DKA in children without COVID-19 infection. As a special type of fulminant T1D, Flu-like symptoms (fever, upper respiratory symptoms, etc.) are frequently observed as preceding symptoms [71]. Besides, some children with DKA presented at the hospital with gastroenterological symptoms, such as nausea, vomiting, diarrhea, and altered mental status [72–74]. Analysis of blood gas results demonstrated a significant decrease in laboratory parameters, including pH and HCO3 lactate. These findings suggest a more severe dysfunction of aqueous electrolytes in children affected by both COVID-19 and DKA [75]. Further laboratory tests conducted by Merav Gil Margolis and colleagues revealed higher HbA1c levels and a lower occurrence of diabetes antibodies in the infection group compared to the COVID-19-negative group [76].

DKA is a severe complication commonly occurring in T1D patients. Its treatment involves several steps, with the International Society of Pediatric and Adolescent Diabetes (ISPAD) recommending fluid and electrolyte replacement as the first step. This helps restore circulating volume, replace sodium and water deficits, and improve glomerular filtration while aiding the clearance of glucose and ketones from the blood. Subsequently, insulin therapy is initiated, followed by the introduction of oral fluids, and transitioning to subcutaneous insulin injections. These decisions are based on monitoring various metrics, including the recovery of serum electrolytes, glucose, blood urea nitrogen, calcium, magnesium, phosphate, and blood gases.

When children with new-onset diabetes exhibit signs of DKA, such as somnolence and polypnea, it’s crucial for physicians to initiate prompt treatment. Beyond the noted heightened insulin requirements mentioned in the study, the fundamental treatment strategies and post-treatment outcomes did not exhibit significant differences from those children suffering DKA without infection [77]. Amid the pandemic, given the constraints on intensive care unit (ICU) clinical resources and blood glucose monitoring devices, reevaluating DKA treatment protocols becomes imperative. While subcutaneous insulin therapy was not traditionally employed for DKA treatment pre-pandemic, emerging clinical trials suggest its efficacy for adults with mild to moderate DKA [78, 79]. In practical terms, initiating treatment with a subcutaneous rapid-acting insulin analog at a dosage of 0.15 U/kg post-fluid replacement is recommended. Subsequent dosage adjustments every two hours should be guided by continuous blood glucose monitoring [80]. Additionally, subcutaneous short-acting regular insulin stands as a viable and safe alternative for pediatric patients with mild to moderate DKA. A starting dose of 0.13 to 0.17 U/kg every four hours, adjustable based on blood glucose levels, is advised. In situations where patient tissue perfusion remains compromised, intramuscular insulin administration is advocated [80].

In the present era, there are numerous innovative devices designed for monitoring glucose levels, and these advancements hold significant potential, especially during the pandemic. Despite the widespread use of glucometers as a popular tool for monitoring glucose levels, they fall short in providing real-time results to comprehend the entire trend in the DKA process [60]. HbA1c levels, often used as a long-term monitoring substitute, may not promptly identify glucose abnormalities during significant changes. Continuous Glucose Monitoring (CGM) devices are increasingly becoming commonplace in managing diabetes patients [81]. These devices can capture and measure blood glucose levels in real time [82]. Scientists have observed that CGM data accurately reflects the fluctuations and baseline levels of glucose in the blood, aiding in determining insulin doses and ensuring glucose safety [83]. An advanced device that integrates the detection of blood glucose and an insulin pump can adjust insulin doses based on routine CGM levels [84]. However, due to the characteristics of high cost, complexity, and need for expertise, CGM could serves as a valuable supplement and should not replace point-of-care blood glucose testing for children [85].

### Prospects of research between COVID-19 and diabetes

Based on bi-directional relationships between diabetes and COVID-19, it is vital to figure out whether diabetes is a consequence of COVID-19 or the complication of virus infection. In this case, more evidence from epidemiology research or long-time follow-up study are needed to support the idea. In reviews and comments, many scientists mentioned the better outcome in patients injection vaccines [86]. As a complication of COVID-19 infection, incidence of diabetes could be inferred to be lower after vaccine injection, however, the solid evidence is still needed. Besides, diabetes is a systematic disease based on the dysfunction of endocrinology organs and tissues, more studies should be conducted to figure out the role of liver and adipose tissue in the incidence and process of new-onset diabetes after infection. Furthermore, with the research on the mechanisms of diabetes, more spot-light targets and fields has been revealed and raised the attention, including microbiome and Inheritance [87, 88]. And these new-found pathways could be the bridges between diabetes and virus infection. In conclusion, the aim and scope of investigating the mechanisms behind the relationships was to develop pharmaceutical interventions to ameliorate infection-related dysglycemia and broad the boundary of diabetes prevention and treatment.

However, the review also has some limitations. Firstly, the narrative review itself has inherent limitations. To mitigate bias in literature selection and evaluation, it would be beneficial to apply systematic review or meta-analysis to draw definitive conclusions regarding the relationships between infection and new-onset diabetes. Additionally, as basic research on the infection of COVID-19 and diabetes advances, our understanding of these two diseases will deepen, leading to the discovery of more pathways and correlations that support the causal relationships between them. Furthermore, as an increasing number of cases involving children with virus infections and diabetes are reported, a comprehensive and reliable description of clinical features will be summarized. This will help derive effective measures to prevent the incidence of diabetes after viral infections, not restricted to COVID-19.

## Conclusion

During the COVID-19 pandemic, there has been an increase in the incidence of new-onset diabetes, particularly with a higher prevalence of Diabetic Ketoacidosis (DKA) and severe DKA. Both in vivo and in vitro basic research provide multiple mechanisms to elucidate the risk of diabetes following a COVID-19 infection. Considering COVID-19 as a type of virus, the pandemic has imparted valuable experiences and lessons. By applying these insights, we can significantly improve our prevention and treatment strategies for virus-induced new-onset diabetes. This, in turn, will help reduce patient mortality rates and alleviate the economic burden on both individual countries and the global community.

## Data Availability

No datasets were generated or analysed during the current study.
